# Valley splitting and anomalous valley Hall effect in MoTe_2_/CrSCl heterostructure

**DOI:** 10.1039/d5na00834d

**Published:** 2025-12-05

**Authors:** Jaehong Park, Dongchul Sung, Junho Yun, Suklyun Hong

**Affiliations:** a Department of Physics, Graphene Research Institute, Quantum Information Science and Technology Center, and KUU Quantum Materials·Devices International Research Center, Sejong University Seoul 05006 Korea hong@sejong.ac.kr

## Abstract

Two-dimensional valleytronics offers a promising platform for novel information processing and quantum technologies by harnessing the valley degree of freedom. A key challenge lies in lifting valley degeneracy, for which magnetic proximity effects provide a promising route. Here, we demonstrate substantial valley splitting in a MoTe_2_/CrSCl heterostructure *via* first-principles calculations. We show that interlayer charge transfer and interfacial orbital hybridization critically govern the valley physics at the interface. Under a moderate in-plane tensile strain (3%) and an applied out-of-plane electric field (0.2 V Å^−1^), a sizable valley splitting of 63 meV emerges at the valence band maximum. These conditions induce hole doping and a pronounced Berry curvature of −23 Å^2^ at the K valley, realizing an electrically tunable anomalous valley Hall effect. Our findings establish the MoTe_2_/CrSCl interface as a versatile platform for valley-selective charge transport, opening pathways for valleytronic device applications.

## Abstract 2

Two-dimensional valleytronics offers a promising platform for novel information processing and quantum technologies by harnessing the valley degree of freedom.

## Graphical abstract

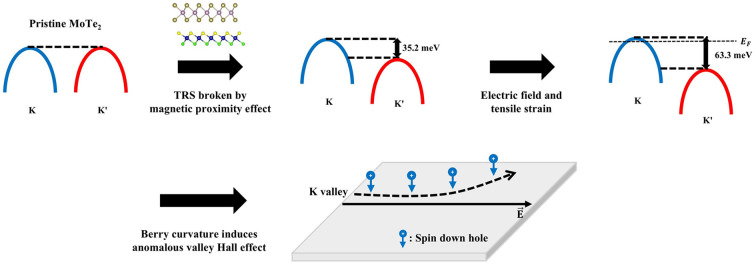

## 1 Introduction

Valleys, which are local extrema in the electronic band structures of two-dimensional materials, provide a new degree of freedom that has led to the field of valleytronics.^1–4^ The valley index holds great potential for applications in information processing and quantum computing.^5–7^ In this context, monolayer transition metal dichalcogenides (TMDs; MX_2_, where M = Mo or W and X = S or Se) have attracted considerable attention. Due to their lack of inversion symmetry, TMDs exhibit two degenerate but inequivalent valleys located at the *K* and *K*′ points of the Brillouin zone.^8^ Furthermore, the strong spin–orbit coupling in TMDs gives rise to spin splitting at these valleys, and due to time-reversal symmetry the spin orientations at the *K* and *K*′ valleys point in opposite directions.^9^ This intrinsic spin–valley coupling in monolayer TMDs makes them highly promising candidates for valleytronic device applications.^10–13^

A major challenge in utilizing the valley degree of freedom lies in lifting the valley degeneracy between the *K* and *K*′ points in the band structure of TMD materials. Since valley degeneracy is protected by time-reversal symmetry, breaking this symmetry is essential for valley manipulation. This can be achieved through two main approaches: optical and magnetic methods. First, the optical approach involves the use of circularly polarized light, which can selectively excite carriers in either the *K* or *K*′ valley depending on the light's polarization.^14–17^ However, this method faces practical limitations due to the need for precise control and the short carrier lifetime resulting from rapid recombination. Second, the magnetic approach provides a more robust means to break time-reversal symmetry and induce valley splitting. This category can be further divided into four strategies. (i) The application of an external magnetic field is a straightforward method,^18–21^ but the resulting valley splitting is relatively small (∼0.1–0.2 meV T^−1^), requiring extremely strong fields for practical use. (ii) Magnetic doping, where magnetic atoms are introduced into the TMD lattice, can generate local magnetic moments that interact with valley states. However, this often leads to the formation of impurity states, which significantly degrade carrier mobility and transport properties.^22–24^ (iii) A promising alternative is the construction of van der Waals (vdW) heterostructures with magnetic materials, which leverages the magnetic proximity effect to induce sizable valley splitting without introducing impurities.^25,26^ (iv) Ferrovalley materials, which possess intrinsic ferromagnetism and valley properties. In such systems, spontaneous valley polarization occurs even without external magnetic fields or doping, as in monolayer 2H-VSe_2_.^27^ Extensive theoretical and experimental efforts have therefore focused on the magnetic approach, particularly the realization of magnetic proximity effects in TMD-based heterostructures. Such systems combine nonmagnetic TMD layers with magnetic substrates such as EuO,^28,29^ EuS,^30,31^ MnO,^32^ CoO,^33^ and CrI_3_.^34–37^ For example, studies on WS_2_/EuS heterostructures have shown that the induced valley splitting decreases with increasing temperature and vanishes above the Curie temperature (*T*_c_) of EuS. This underscores the need for high-*T*_c_ magnetic materials that can maintain valley splitting at elevated temperatures for practical valleytronic applications.

Monolayer CrSCl has recently been predicted to be a ferromagnetic semiconductor with a magnetic moment of 3 µ_B_ and a Curie temperature of 273 K.^38,39^ These properties make monolayer CrSCl a promising candidate as a magnetic layer in vdW heterostructures with TMDs, enabling the use of magnetic proximity effects to achieve robust and controllable valley splitting. Notably, monolayer CrSCl exhibits a Janus-type structure with asymmetric S and Cl atomic planes, leading to an intrinsic out-of-plane dipole and distinct top and bottom chemical environments. Such structural asymmetry can produce different valley and spin polarizations depending on the contact side, as demonstrated in other Janus and ferroelectric systems.^40–42^

In this work, we explore the magnetic proximity effect as a means to lift valley degeneracy in two-dimensional materials. To this end, we construct a MoTe_2_/CrSCl heterostructure and carry out density functional theory (DFT) and Berry curvature calculations to uncover the underlying mechanisms of valley splitting and valley-polarized transport. The intrinsic magnetic and structural properties of monolayer CrSCl make it a compelling substrate for proximity-induced valley manipulation. Owing to its structural asymmetry, the interface offers two different stacking configurations, enabling detailed investigation of interfacial interactions. Our study reveals that interlayer charge transfer and orbital hybridization play key roles in determining the extent of valley splitting, and that the symmetry breaking at the interface gives rise to nontrivial Berry curvature distributions leading to valley-polarized transport, relevant to valleytronic applications.

## 2 Computational details

We performed first-principles calculations using the Vienna *Ab Initio* Simulation Package (VASP),^43,44^ incorporating the generalized gradient approximation (GGA) of the Perdew–Burke–Ernzerhof (PBE) functional.^45^ Ion-electron interactions were described using the projector augmented wave (PAW) method,^46,47^ and the van der Waals interaction was taken into consideration using the Grimme's DFT-D3 method.^48^ A plane-wave basis set with a cut-off energy of 500 eV was used to expand the electronic wave functions. For Brillouin-zone sampling, a *Γ*-centered 18 × 18 × 1 k-mesh was used. To consider the correlation effects in the Cr-3d orbital, the DFT + U method was applied with *U* − *J* = 2.1 eV.^38,39^ The convergence criteria were set to 0.001 eV Å^−1^ for forces and 10^−8^ eV for energy. Spin–orbit coupling (SOC) was included in all electronic structure calculations, and a vacuum space of 20 Å was introduced along the *z* direction. For the calculation of the Berry curvature, VASPBERRY^49^ code was used.

## 3 Result and discussion

### 3.1 Optimized MoTe_2_/CrSCl heterostructures and their stability

**Fig. 1:**
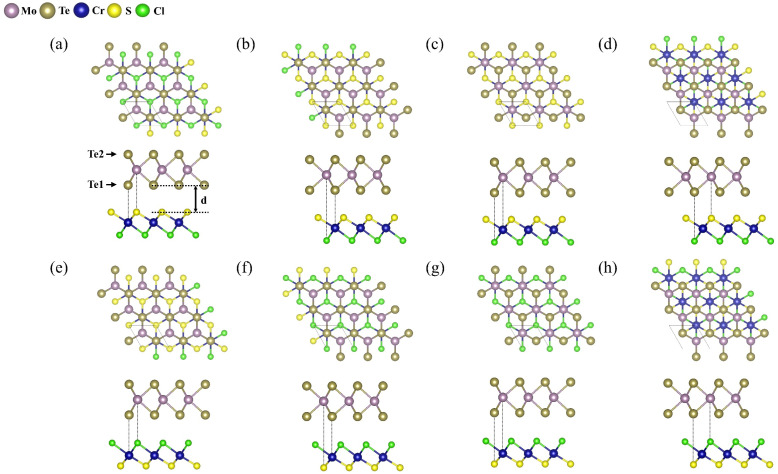
Top and side views of MoTe_2_/CrSCl heterostructures in eight stacking configurations. (a) S-1 (b) S-2 (c) S-3 (d) S-4 (e) Cl-1 (f) Cl-2 (g) Cl-3 (h) Cl-4. The interlayer distance is denoted as *d*.

First, we optimize the geometries of the vdW MoTe_2_/CrSCl heterostructures. The lattice constants of monolayer CrSCl and MoTe_2_ are 3.453 Å and 3.518 Å, respectively, resulting in a small lattice mismatch of 1.80%, which allows the construction of a commensurate 1 × 1 heterostructured supercell. To explore the interfacial properties, we consider eight possible stacking configurations of MoTe_2_/CrSCl heterostructure, labeled S-1 to S-4 and Cl-1 to Cl-4, depending on whether the S or Cl layer of CrSCl faces the Te atoms of MoTe_2_ at the interface (see Fig. 1).

In S-1 and Cl-1, Te1 atoms are located above Cr atoms, while Mo atoms are above S (in S-1) or Cl (in Cl-1) atoms. In S-2 and Cl-2, Te1 atoms are above Cr atoms, and Mo atoms are above Cl (in S-2) or S (in Cl-2) atoms. In S-3 and Cl-3, Mo atoms are placed above Cr atoms, while Te1 atoms are above Cl (in S-3) or S (in Cl-3) atoms. In S-4 and Cl-4, Mo atoms are above S (in S-4) or Cl (in Cl-4) atoms, and Te1 atoms are above Cl (in S-4) or S (in Cl-4) atoms. In configurations S-2, S-4, Cl-2, and Cl-4, the MoTe_2_ layer is rotated by 180°, introducing structural asymmetry. Detailed atomic structures are presented in Fig. 1, and further information on each configuration is summarized in Table 1.

**Table 1:** Comparison between the different stacking configurations. Here, *a*_0_ denotes the optimized lattice constant, *E*_b_ is the binding energy, *d* is the interlayer distance, and Δ_*KK***′**_represents the valley splitting at the valence band maximum (VBM) between the *K* and *K*′ points

Configuration	S-1	S-2	S-3	S-4	Cl-1	Cl-2	Cl-3	Cl-4
*a*_0_ (Å)	3.492	3.494	3.496	3.493	3.491	3.491	3.492	3.491
*E*_b_ (eV)	−0.28	−0.27	−0.28	−0.29	−0.22	−0.22	−0.22	−0.22
*d* (Å)	3.21	3.26	3.17	3.12	3.33	3.35	3.37	3.33
Δ_*KK***′**_ (meV)	27.9	35.2	33.3	6.9	4.1	5.3	6.7	0.5

To evaluate the relative stability of the different configurations, their binding energies are calculated. The binding energy *E*_b_ of each heterostructure is defined by *E*_b_ = *E*_MoTe_2_/CrSCl_ − *E*_MoTe_2__ − *E*_CrSCl_, where *E*_MoTe_2_/CrSCl_, *E*_MoTe_2__, *E*_CrSCl_ are the total energies of the heterostructure, monolayer MoTe_2_, and monolayer CrSCl, respectively. As shown in Table 1, all stacking configurations exhibit negative binding energies, indicating that they are thermodynamically stable. Notably, the S-side configurations consistently show lower binding energies than the Cl-side ones, suggesting that the S-interface structures are energetically more favorable.

### 3.2 Origin of the difference in valley splitting magnitude

**Fig. 2:**
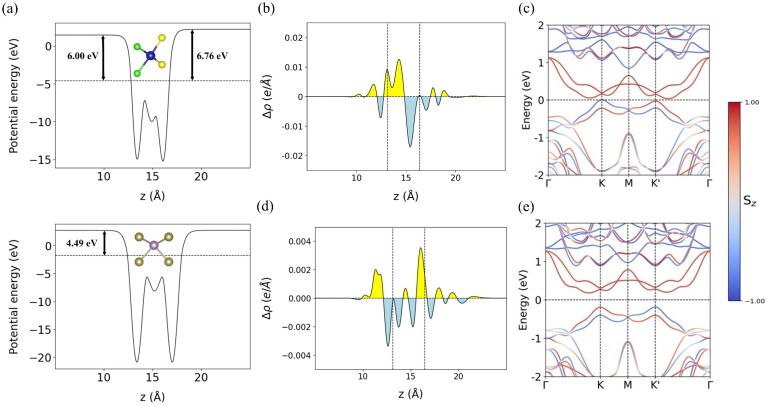
(a) Top and bottom panels represent the electrostatic potential energy of monolayer CrSCl and monolayer MoTe_2_, respectively. The black arrows indicate work functions. (b) and (c) represent the charge density difference and band structure of the S-2 configuration, respectively, while (d) and (e) indicate the corresponding results for Cl-3. The Fermi level is set to zero. The color bar (*S*_*z*_) indicates the spin-*z* expectation value, where positive (negative) values correspond to spin-up (spin-down) components.

The valley splitting of valence band maximum (VBM), denoted as Δ_*KK***′**_, is defined as the energy difference between the VBMs at the *K* and *K*′ points. Overall, the S-side configurations exhibit larger valley splitting than their Cl-side counterparts. For detailed analysis, we select the configurations with the largest Δ_*KK***′**_ values from each group: S-2 (35.2 meV) and Cl-3 (6.7 meV). Although S-2 is not the most stable among the S-side configurations, it is chosen for further investigation due to its prominent valley splitting. The calculated band structures for S-2 and Cl-3, obtained with spin–orbit coupling calculations, are shown in Fig. 2(c) and (e), respectively.

To investigate the origin of the difference in valley splitting magnitude, we compare the work functions of monolayer MoTe_2_ and monolayer CrSCl, as well as the charge density differences in the S-2 and Cl-3 heterostructures, as illustrated in Fig. 2.

The work function of monolayer CrSCl depends on the surface termination, with values of 6.00 eV for the Cl-terminated side and 6.76 eV for the S-terminated side. In contrast, monolayer MoTe_2_ has a work function of 4.49 eV, as shown in Fig. 2(a). Since the work function of CrSCl is higher than that of MoTe_2_ on both surfaces, electrons are transferred from MoTe_2_ to CrSCl at the interface. Fig. 2(b) and (d) show the calculated charge density differences for the S-2 and Cl-3 configurations, respectively. A more pronounced charge redistribution is observed at the interface in the S-2 configuration compared to Cl-3. To quantify the interlayer charge transfer, we employ Bader charge analysis,^50^ revealing that 0.037 electrons are transferred from MoTe_2_ to CrSCl in S-2, whereas only 0.018 electrons are transferred in Cl-3. This difference can be attributed to the high electronegativity of Cl atoms, which tend to attract more charge than S atoms. As a result, stronger electron repulsion occurs on the Cl-terminated side, inhibiting interlayer charge transfer in Cl-3 compared to S-2.^51^ These results indicate that interlayer charge transfer plays a crucial role in determining the magnitude of valley splitting. This also explains why the Cl-side stacking is energetically less favorable and exhibits a larger interlayer distance: all Cl-side configurations already have more accumulated charge on the interfacial Cl atoms, which suppresses further charge transfer and weakens the interlayer coupling.

**Fig. 3:**
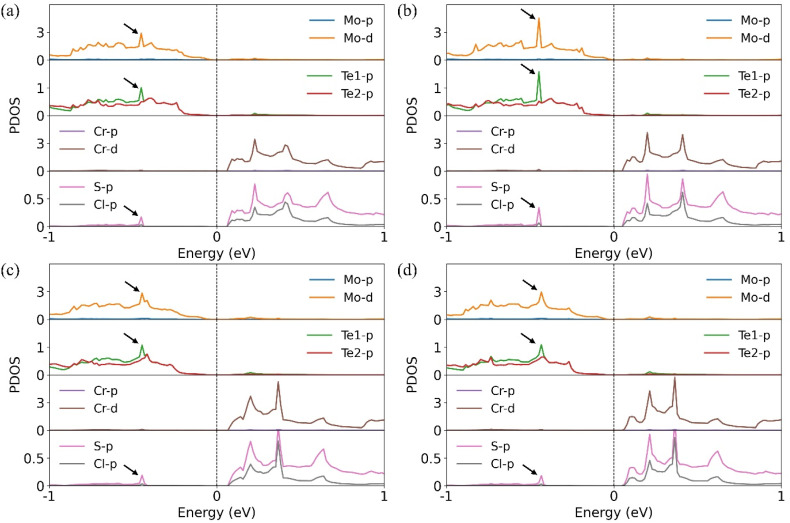
Projected density of states (PDOS) for each configuration: (a) S-1 (b) S-2 (c) S-3 and (d) S-4. The Fermi level is set to zero.

To further understand why S-2 exhibits the largest valley splitting among the S-side configurations, we first analyze the interlayer charge transfer in each case. The calculated charge transfer amounts for S-1 through S-4 are 0.039 e^−^, 0.037 e^−^, 0.042 e^−^, and 0.042 e^−^, respectively. Interestingly, S-2 shows the smallest interlayer charge transfer, despite exhibiting the largest valley splitting. To clarify this counterintuitive result, we examine the projected density of states (PDOS) for each configuration, as shown in Fig. 3. In S-2, strong hybridization occurs between Mo, Te1, and S atoms at the interface (Fig. 3(b)), resulting in the largest valley splitting. By contrast, S-1, S-3, and S-4 exhibit weaker hybridization (Fig. 3(a), (c), and (d)) and smaller valley splitting compared to S-2. In addition, in all cases, the VBM originates from the MoTe_2_ layer, while the conduction band minimum (CBM) is mainly contributed by the CrSCl layer. This confirms that all heterostructures exhibit staggered (type-II) band alignment,^52^ resulting in spatial separation of electrons and holes.

These results suggest that both interlayer charge transfer and interfacial orbital hybridization contribute to the magnitude of valley splitting, with the latter playing a particularly important role in the case of S-2. In addition, S-4 and Cl-4 exhibit the weakest splitting, as their Mo atomic positions are not in good spatial superposition, that is, not vertically aligned with the magnetic Cr atoms.^35^

### 3.3 Tunable valley splitting *via* electric fields and strains

**Fig. 4:**
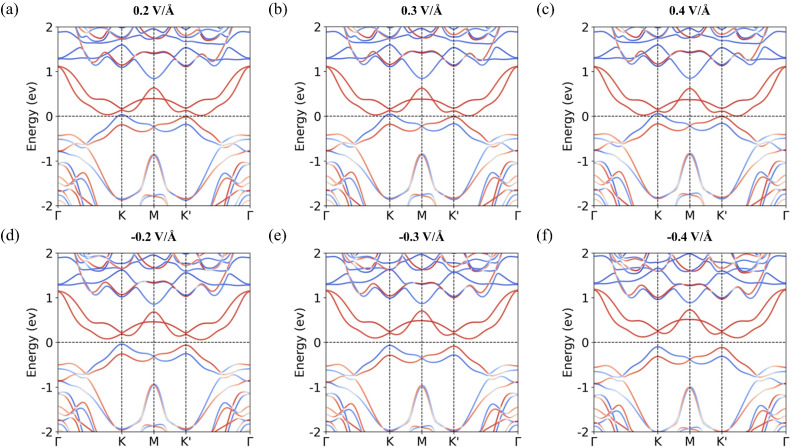
Calculated band structures of the S-2 configuration under external electric fields: (a) 0.2 V Å^−1^, (b) 0.3 V Å^−1^, (c) 0.4 V Å^−1^, (d) −0.2 V Å^−1^, (e) −0.3 V Å^−1^, and (f) −0.4 V Å^−1^.

Next, we explore strategies for tuning the magnitude of valley splitting. Although the binding energies of all stacking configurations are quite similar, a mixture of different configurations may exist in experiments. Nevertheless, we focus on the S-2 configuration because it shows the largest valley splitting, making it the most representative case for exploring the tunability of valley splitting. As mentioned above, the valley splitting is closely related to the extent of interlayer charge transfer. External electric fields and biaxial strain can effectively tune the band alignment of the heterostructure, thereby modulating the interlayer charge transfer.^53–55^ To examine this effect, we first apply an out-of-plane (*z*-direction) electric field since the charge transfer from MoTe_2_ to CrSCl occurs predominantly along this direction. The resulting band structures under various electric field strengths are shown in Fig. 4. To enhance the charge transfer from MoTe_2_ to CrSCl, a positive electric field is applied. This leads to increased charge transfer, causing the CBM and VBM to move closer together, thereby enhancing the valley splitting, as shown in Fig. 4(a)–(c). In contrast, applying a negative electric field reduces the charge transfer from MoTe_2_ to CrSCl. This results in greater separation between the CBM and VBM and a corresponding decrease in valley splitting, as shown in Fig. 4(d)–(f). Table 2 summarizes the calculated charge transfer amounts and corresponding valley splitting values under various electric field conditions.

**Table 2:** Comparison of different electric field conditions for the S-2 configuration. Charge transfer occurs from MoTe_2_ to CrSCl. Δ_*KK***′**_ represents the valley splitting at the VBMs at the *K* and *K*′ points

Electric field	−0.4 V Å^−1^	−0.3 V Å^−1^	−0.2 V Å^−1^	0 V Å^−1^	0.2 V Å^−1^	0.3 V Å^−1^	0.4 V Å^−1^
Charge transfer (e^−^)	0.02	0.024	0.028	0.037	0.048	0.053	0.059
Δ_*KK***′**_ (meV)	14.5	18.5	23.4	35.2	50.4	58.9	65.6

**Fig. 5:**
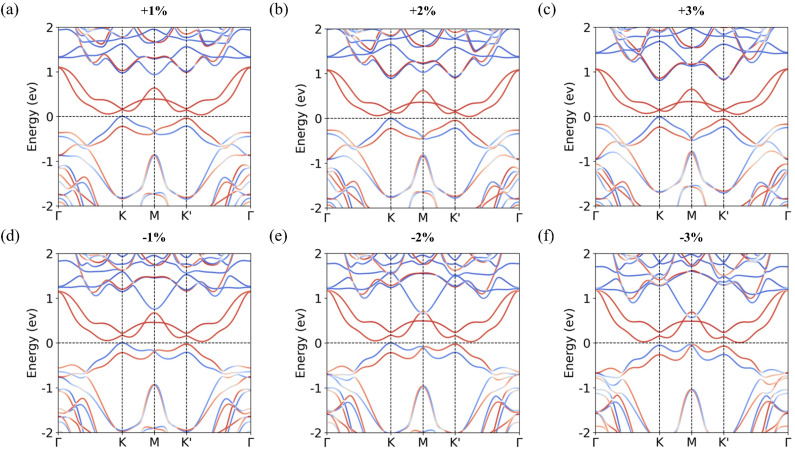
Calculated band structures for the S-2 configuration with in-plane biaxial strain. (a) *ε* = 1%, (b) *ε* = 2%, (c) *ε* = 3%, (d) *ε* = −1%, (e) *ε* = −2%, and (f) *ε* = −3%.

Likewise, in-plane biaxial strain can also modulate the interlayer coupling and hence the valley splitting.^32^ The biaxial strain is defined as
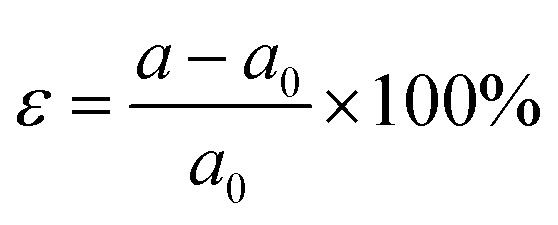
where *a*_0_ and *a* denote the unstrained and strained lattice constants, respectively. A positive value (*ε* > 0) corresponds to tensile strain, while a negative value (*ε* < 0) corresponds to compressive strain. The calculated band structures under biaxial strain are presented in Fig. 5. Under tensile strain, the valley splitting increases slightly, accompanied by an upward shift of the VBM at the *Γ* point [Fig. 5(a)–(c)]. In contrast, compressive strain leads to a slight reduction in valley splitting, while the VBM at the M point shifts upward [Fig. 5(d)–(f)].

To understand these variations, we analyze the interlayer distance and the amount of interlayer charge transfer, as summarized in Table 3. When tensile strain is applied, the interlayer distance decreases, thereby strengthening interlayer coupling.^56^ This stronger coupling promotes greater interlayer charge transfer, resulting in an increase in valley splitting. Conversely, the interlayer distance increases under compressive strain, the interlayer coupling strength becomes weaker, leading to reduced charge transfer and a corresponding decrease in valley splitting. Notably, under 3% compressive strain [Fig. 5(f)], the VBM at the M point surpasses that at the *K* and *K*′ points, causing the valley characteristics of the MoTe_2_/CrSCl heterostructure to disappear. Furthermore, a previous study has reported that in-plane tensile strain can enhance the Curie temperature (*T*_c_) of monolayer CrSCl.^38^ Therefore, applying in-plane tensile strain serves as an effective approach to simultaneously enhance both the valley splitting and the Curie temperature in the MoTe_2_/CrSCl heterostructure.

**Table 3:** Comparison of different biaxial strain conditions for the S-2 configuration. *d* denotes the interlayer distance. Charge transfer occurs from MoTe_2_ to CrSCl. Δ_*KK***′**_ represents the valley splitting at VBMs at the *K* and *K*′ point

Strain	−3%	−2%	−1%	0%	+1%	+2%	+3%
*d* (Å)	3.33	3.3	3.28	3.26	3.23	3.2	3.18
Charge transfer (e^−^)	0.033	0.034	0.036	0.037	0.039	0.041	0.043
Δ_*KK***′**_ (meV)	18.6	25.7	30.2	35.2	39.5	43.2	43.2

The overall trends of the valley splitting and interlayer charge transfer at the VBMs at the *K* and *K*′ point, as functions of the applied electric field and biaxial strain, are illustrated in Supplementary Fig. S1.

### 3.4 Anomalous valley Hall effect

In the MoTe_2_/CrSCl heterostructure, the breaking of both inversion symmetry and time-reversal symmetry gives rise to a nonzero Berry curvature at the *K* and *K*′ points, with opposite signs and differing magnitudes. The Berry curvature for all occupied states, derived from the Kubo formula, is defined by^57,58^
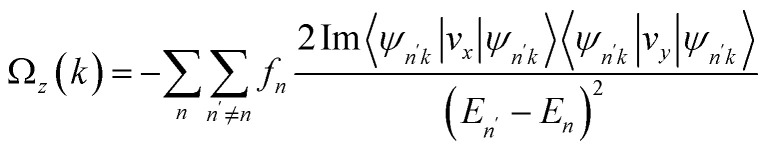
where *f*_*n*_ is the Fermi-Dirac distribution function, *v*_*x*_ and *v*_*y*_ are the velocity operators, and *ψ*_*nk*_ is the Bloch wavefunction associated with eigenvalue *E*_*n*_. This Berry curvature induces an anomalous velocity of carriers at the K and K′ valleys, expressed as *v* ∼ *E* × Ω_*z*_(*k*),^59^ where *E* and Ω_*z*_(*k*) are the applied in-plane electric field and Berry curvature, respectively. When the Fermi level lies between the VBM of the *K* and *K*′ valleys, hole doping occurs only in the valley with the higher VBM energy. These carriers acquire an anomalous transverse velocity, resulting in valley-polarized transport, a phenomenon known as the anomalous valley Hall effect.^27^

**Fig. 6:**
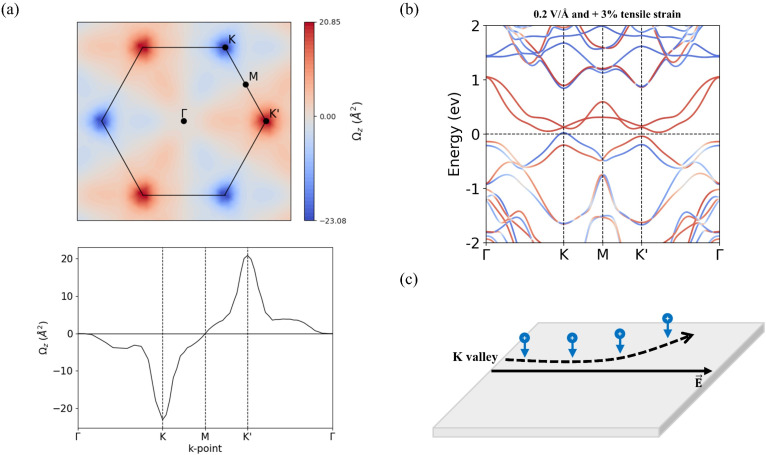
Under 0.2 V Å^−1^ electric field and 3% in-plane tensile strain applied to the S-2 configuration: (a) contour map of the Berry curvature in the 2D Brillouin zone and the Berry curvature along a high-symmetry path; (b) calculated band structure; (c) schematic illustration of the anomalous valley Hall effect. The blue down-arrow represents a spin-down hole.

To achieve a pronounced anomalous valley Hall effect and further enhance valley splitting, we apply a combination of a 0.2 V Å^−1^ out-of-plane electric field and 3% in-plane biaxial tensile strain—two modulation strategies previously shown to be effective. The resulting band structure, shown in Fig. 6(b), reveals a substantial valley splitting of 63.3 meV, along with hole doping in the K valley. Fig. 6(a) illustrates the Berry curvature under these conditions as a contour map in the 2D Brillouin zone and along a high-symmetry path, respectively. The Berry curvature at the *K* valley reaches −23.08 Å^2^. When an in-plane electric field is applied, spin-down hole carriers at the *K* valley acquire an upward anomalous velocity and accumulate along one side of the sample, as schematically depicted in Fig. 6(c). This results in a spin- and valley-dependent Hall voltage.

In realistic conditions, however, the Fermi level may not be precisely located between the two valleys. Even in such cases, a measurable transverse valley Hall voltage can still arise because the Berry curvature magnitudes at the *K* (23.08 Å^2^) and *K*′ (20.85 Å^2^) valleys are not identical. This asymmetry produces unequal anomalous velocities for carriers in the two valleys, leading to a net valley Hall voltage and maintaining spin-polarized transport due to the intrinsic spin–valley locking of the heterostructure.^60^

Under these combined conditions, the MoTe_2_/CrSCl heterostructure exhibits both a pronounced anomalous valley Hall effect and a high Curie temperature, making it a promising candidate for next-generation, high-performance valleytronic applications.

## 4 Conclusion

In summary, we conducted a comparative study of MoTe_2_/CrSCl heterostructures using DFT calculations to investigate the key factors affecting valley splitting. Our results reveal that interlayer charge transfer and interfacial orbital hybridization play critical roles in determining the magnitude of valley splitting. To further control the magnitude of the valley splitting, we applied both an external electric field and in-plane biaxial strain, each of which effectively influences the interlayer charge transfer. Under a positive electric field, the valley splitting increased significantly, while a slight increase was observed under biaxial tensile strain. When a 0.2 V Å^−1^ electric field and 3% biaxial tensile strain were applied simultaneously, the valley splitting reached 63.3 meV, accompanied by hole doping at the *K* point. This hole occupation at the *K* valley facilitates valley-polarized transport. These findings suggest that the MoTe_2_/CrSCl heterostructure is a promising candidate for future valleytronic device applications.

## Author contributions

Jaehong Park: conceptualization, investigation, formal analysis, visualization, data curation, writing – original draft. Dongchul Sung: formal analysis, writing – review and editing. Junho Yun: formal analysis. Suklyun Hong: conceptualization, investigation, formal analysis, supervision, writing – review and editing, resources, funding acquisition.

## Conflicts of interest

There are no conflicts to declare.

## Supplementary Material

NA-OLF-D5NA00834D-s001

## Data Availability

The structure data for each stacking configuration can be found at https://doi.org/10.5281/zenodo.16760791. Supplementary information (SI) is available. See DOI: https://doi.org/10.1039/d5na00834d.
