# Supplementary material for: Valley splitting and anomalous valley Hall effect in MoTe2/CrSCl heterostructure
Source: Nanoscale Adv. 2025 Dec 5;8(2):580–7. doi: 10.1039/d5na00834d (PMC12679478; doi:10.1039/d5na00834d)
Supplement: NA-008-D5NA00834D-s001 [file NA-008-D5NA00834D-s001.pdf]

Supplementary Information (SI) for Nanoscale Advances.
This journal is © The Royal Society of Chemistry 2025

Supplementary Information

Valley splitting and anomalous valley Hall effect in $MoTe_2$/CrSCl heterostructure

Jaehong Park[a], Dongchul Sung[a], Junho Yun[a], and Suklyun Hong[a,*]

[a] Department of Physics, Graphene Research Institute, Quantum Information Science and Technology Center, and KUU Quantum Materials·Devices International Research Center, Sejong University, Seoul 05006, Korea
*Corresponding author: hong@sejong.ac.kr

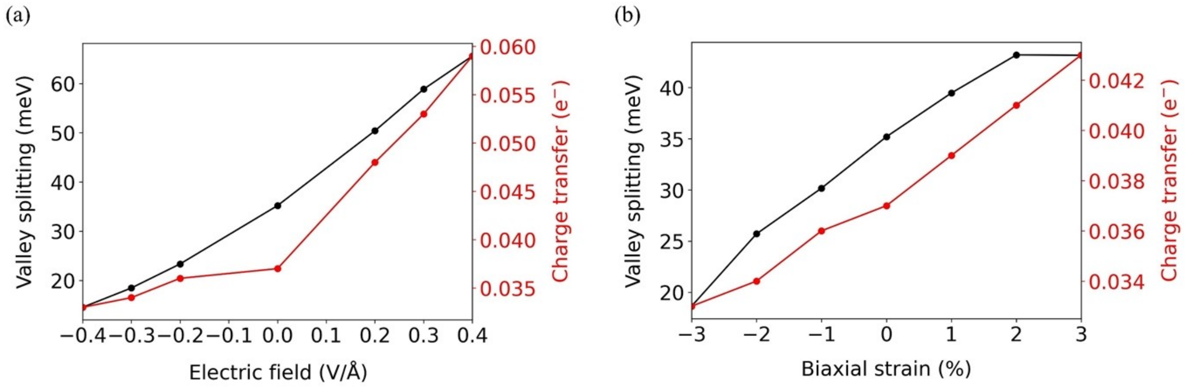

Fig. S1 Valley splitting (black line) and interlayer charge transfer (red line) as functions of (a) the applied electric field and (b) the in-plane biaxial strain.
